# ﻿*Johnstonellapunensis* (Boraginaceae), a new species endemic to the dry Puna of Chile

**DOI:** 10.3897/phytokeys.197.84833

**Published:** 2022-05-30

**Authors:** Michael G. Simpson, Kristen Hasenstab-Lehman, Makenzie E. Mabry, Mélica Muñoz-Schick

**Affiliations:** 1 Department of Biology, San Diego State University, San Diego, California 92182, USA San Diego State University San Diego United States of America; 2 Department of Conservation and Research, Santa Barbara Botanic Garden, Santa Barbara, California 93105, USA Department of Conservation and Research, Santa Barbara Botanic Garden Santa Barbara United States of America; 3 Florida Museum of Natural History, University of Florida, Gainesville, Florida 32611, USA University of Florida Gainesville United States of America; 4 Sección Botánica, Museo Nacional de Historia Natural, Casilla 787, Santiago, Chile Sección Botánica, Museo Nacional de Historia Natural Santiago Chile

**Keywords:** Boraginaceae, Chile, *Cryptantha*, *Johnstonella*, phylogenetics, Puna, taxonomy

## Abstract

In an earlier molecular phylogenetic study, a sample of what was originally identified as *Cryptanthahispida* (Boraginaceae) from Chile, grouped with species of the genus *Johnstonella*. This sample was subsequently shown not to be *C.hispida*, but an undescribed species, endemic to the dry Puna of Chile. This new species is described here as *Johnstonellapunensis*, along with a key to all South American species of the genus. *Johnstonellapunensis* resembles other members of that genus in having an ovate fruit shape, ovate nutlets and a long style that extends beyond the nutlets. It is unusual in the genus in having a non-tuberculate, dimpled to rugulose nutlet surface sculpturing. Its closest relative within the genus is likely the South American *J.diplotricha*.

## Abstract (es)

En un estudio filogenético molecular anterior, una muestra que originalmente se identificó como *Cryptanthahispida* (Boraginaceae) de Chile se agrupaba con especies del género *Johnstonella*. Posteriormente se demostró que esta muestra no era *C.hispida* sino una especie no descrita, endémica de la Puna seca de Chile. Esta nueva especie se describe aquí como *Johnstonellapunensis*, junto con una clave para todas las especies sudamericanas del género. *Johnstonellapunensis* se parece a otros miembros del género por tener un fruto de forma ovadа, clusas ovadas y un estilo largo que sobrepasa las clusas. Es inusual en el género que la clusa tenga una superficie no tuberculada, sino que rugulosa formando hoyuelos. Su pariente más cercano dentro del género es probablemente la especie sudamericana *J.diplotricha*.

## ﻿Introduction

The genus *Johnstonella* Brand (Boraginaceae s. str., after Chacón et al. 2016 and Luebert et al. 2016) was originally segregated from *Cryptantha* Lehmann ex G.Don and described with two species: *Johnstonellainaequata* (I.M.Johnst.) Brand and *J.racemosa* (A.Gray) Brand, the latter the lectotype of the genus (Simpson et al. 2014). The genus was not accepted by subsequent botanists, however, until the molecular phylogenetic study by Hasenstab-Lehman and Simpson (2012). Their Sanger sequencing-based phylogeny inferred a clade distinct from *Cryptantha* and composed of the two species described by Brand (1925), plus another six species formerly classified in *Cryptantha*. Consequently, these eight sequenced species, plus an additional three based on morphology, were transferred from *Cryptantha* to *Johnstonella* by Hasenstab-Lehman and Simpson (2012). (See Simpson et al. 2019 for a complete listing of *Johnstonella* taxa.) Subsequent high-throughput sequencing-based analyses (Simpson et al. 2017a, Mabry and Simpson 2018) confirmed *Johnstonella* to be a well-supported clade, distinct from *Cryptantha*. However, two taxa previously placed in *Johnstonella* by Hasenstab-Lehman and Simpson (2012), based on morphology or Sanger sequencing data – *J.echinosepala* (J.F.Macbride) Hasenstab & M.G.Simpson and *J.micromeres* (A.Gray) Hasenstab & M.G.Simpson – were found to be nested within *Cryptantha*, based on these two later, more comprehensive molecular phylogenetic studies (see also Simpson and Rebman 2021). New nomenclatural combinations by these workers and by Simpson et al. (2019), plus a recent new species discovery by Hinton and Nesom (2021) has led to the current recognition of 16 total species and three varieties in *Johnstonella*, based either on the cited molecular phylogenetic studies or on comparative morphological similarities (Amsinckiinae Working Group 2022).

**Figure 1.**
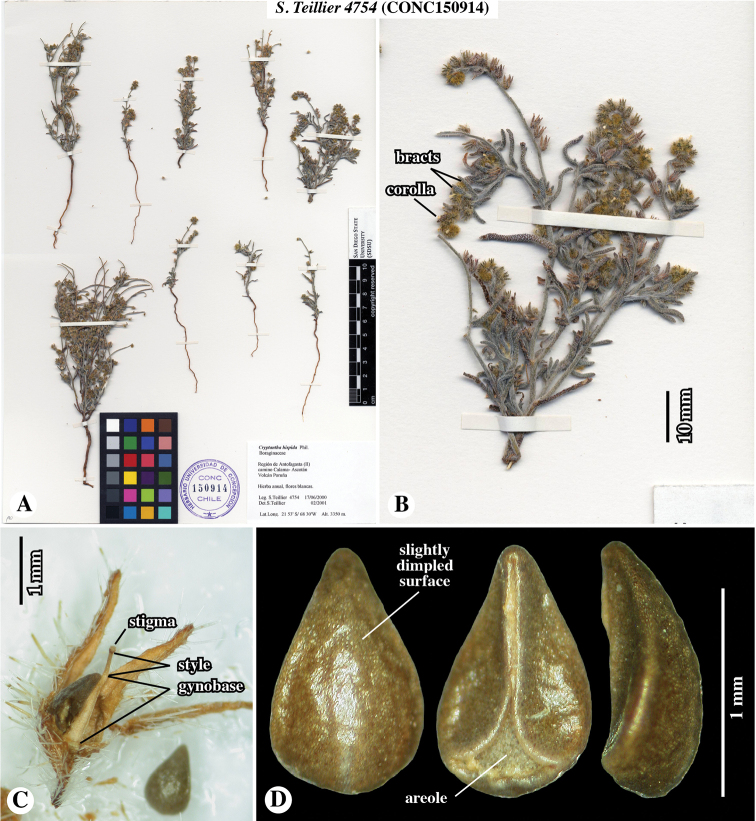
Paratype of *Johnstonellapunensis*, sp. nov., *Teillier 4754* (**CONC**150914) [originally identified as *Cryptanthahispida*] **A** whole herbarium sheet **B** close-up of a single plant; note very small corollas and flower bracts **C** close-up fruit; note dense appressed-strigose along margin and spreading-hispid trichomes along mid-rib **D** nutlet in (left to right) dorsal, ventral and lateral views; note slightly dimpled surface.

In the Simpson et al. (2017a) and Mabry and Simpson (2018) analyses, it was noted that a Chilean species of *Cryptantha* – *C.hispida* (Phil.) Reiche – grouped phylogenetically within *Johnstonella*. This was unexpected given that *Cryptanthahispida* was considered by Johnston (1927), in his revision of the South American *Cryptantha*, to be a close relative of *Cryptanthaphaceloides* (Clos) Reiche. In fact, a specimen of the latter species that was included in Simpson et al. (2017a) grouped with strong support within their “*Cryptantha* s.str.” clade. Examination of the fruit morphology of the sequenced sample of *C.hispida* (*Teillier 4754*; accession number **CONC**150914; see Fig. 1) clearly demonstrated that it had been misidentified because *C.hispida* has a different nutlet morphology (see Simpson et al. 2019 for images of the type of *C.hispida* and further discussion). The specimen involved did, however, resemble *Johnstonella* taxa in features of the calyx and nutlet shape and in style length, but was also distinct from any known species in nutlet sculpturing.

## ﻿Methods

As the *Teillier 4754* (**CONC**150914) sample grouped with strong support phylogenetically within the *Johnstonella* clade in the two cited molecular studies and because of its distinctive nature of nutlet morphology, we thought it a new species. Herbarium specimens at **SGO** were examined in order to ascertain the presence of other specimens of this putative new taxon. Five additional collections that fit the characteristics of the *Teillier 4754* specimen were identified as conspecific. All specimens were studied morphologically and used to create a description and key. Measurements of structures were made with a ruler graduated to 0.1 mm. A type specimen was selected from amongst the **SGO** specimens. Photographic documentation of plant components was made using a Visionary Digital Imaging System photomicroscope or a Nikon Microphot camera attached to an Olympus dissecting microscope. The six total collections of the new taxon were mapped, along with the three other South American species of *Johnstonella*: *J.albida* (Kunth) M.E.Mabry & M.G.Simpson, *J.diplotricha* (Phil.) Hasenstab & M.G.Simpson and *J.parviflora* (Phil.) Hasenstab & M.G.Simpson. The map was created using the BerkeleyMapper tool (https://ucjeps.berkeley.edu/consortium/load_mapper_multi.html) from georeferenced specimen data transcribed or estimated for the six specimens of the new species and from georeferenced specimen data of the other three *Johnstonella* species, the latter derived from records of the Global Biodiversity Information Facility (GBIF.org2022a, b, c). Aspects of biogeographic regions, after Luebert (2021), were overlain on distribution maps. The morphological and biogeographic differences amongst all four *Johnstonella* species were evaluated, the morphology of *J.albida*, *J.diplotricha* and *J.parviflora* being based on study of specimens listed in Appendix 1. All herbarium acronyms after Thiers (2022).

## ﻿Results

Based on comparative morphological studies, we feel confident that the six samples studied represent a new species. In addition, these collections have distributions within the same general range in the Atacama Desert in Chile as the original *Teiller 4754* specimen. We describe the new species as follows.

### ﻿Taxonomic treatment

#### ﻿ Johnstonella punensis

Taxon classificationPlantaeBoraginalesBoraginaceae

M.G.Simpson & Muñoz-Schick sp. nov.

D5C3B658-5E8E-5395-9F52-713914BA739F

urn:lsid:ipni.org:names:77298802-1

**Figure 2.**
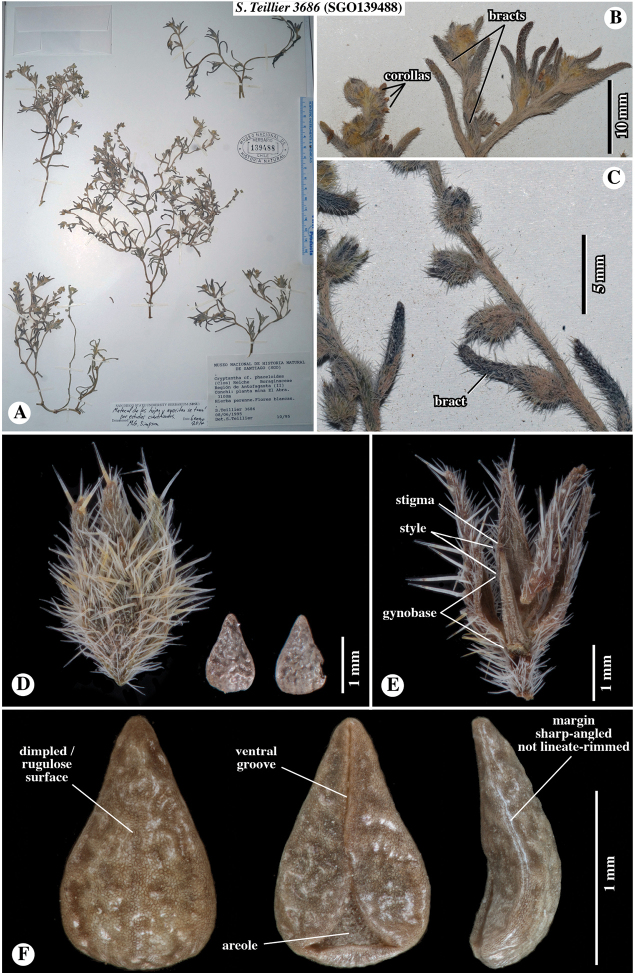
Paratype of *Johnstonellapunensis*, sp. nov., *Teillier 3686* (**SGO**139488) [originally identified as Cryptanthacf.phaceloides] **A** whole herbarium sheet **B** close-up of inflorescences; note very small corollas and flower bracts **C** close-up inflorescence axis and fruits; note dense appressed-strigose and spreading-hispidulous trichomes of axis and note bract subtending lowermost fruit **D** close-up of fruiting calyx, showing short, appressed hirsute trichomes along marginal surface and ascending to horizontal hispid trichomes along mid-rib, only two nutlets per fruit illustrated **E** fruit opened, showing gynobase, style and stigma **F** nutlet, in (left to right) dorsal, ventral and lateral views; note dimpled surface, narrow ventral groove with triangular areole at base and sharp-angled, but not lineate-rimmed margin.

**Figure 3.**
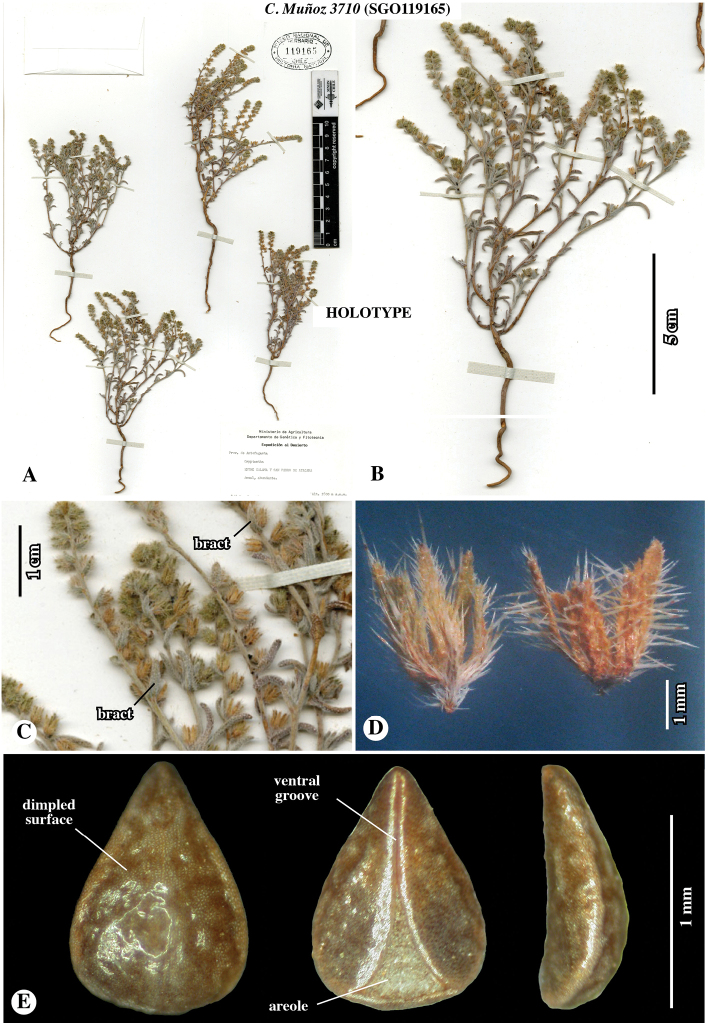
Holotype of *Johnstonellapunensis*, sp. nov., *C. Muñoz 3710* (**SGO**119165) [originally identified as *Cryptantha*] **A** whole herbarium sheet **B** close-up of one plant **C** close-up of inflorescence cymules with fruits; note flower bracts **D** close-up of two fruiting calyces, showing short, appressed to ascending hirsute trichomes along marginal surface and ascending to horizontal hispid trichomes along thickened mid-rib **E** nutlet, in dorsal (left), ventral (middle) and lateral (right) views; note dimpled to rugulose surface and narrow ventral groove with triangular areole at base.

**Figure 4.**
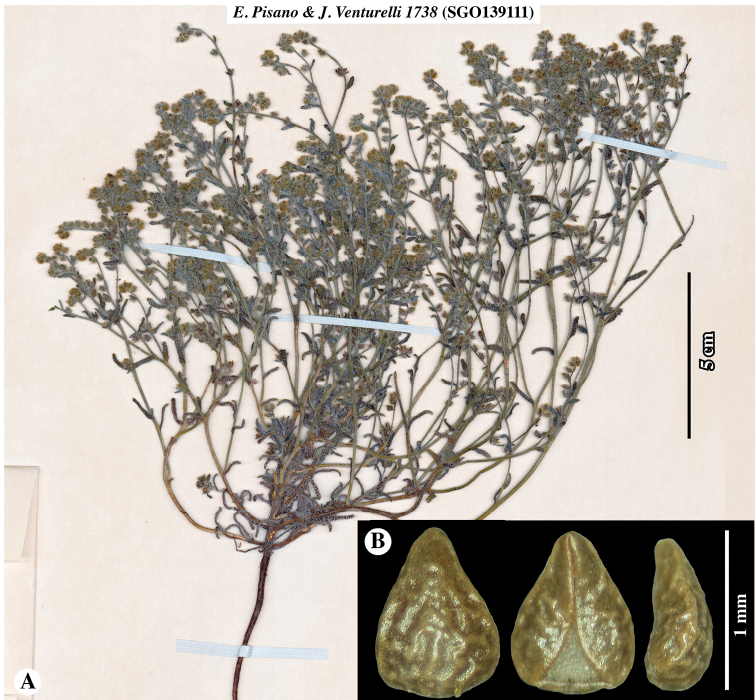
Paratype of *Johnstonellapunensis*, sp. nov., *Pisano & Venturelli 1738* (**SGO**139111) **A** plant from herbarium sheet **B** nutlet, in dorsal (left), ventral (middle) and lateral (right) views; note rugulose surface, narrow ventral groove with triangular areole at base and sharp-angled margin.

**Figure 5.**
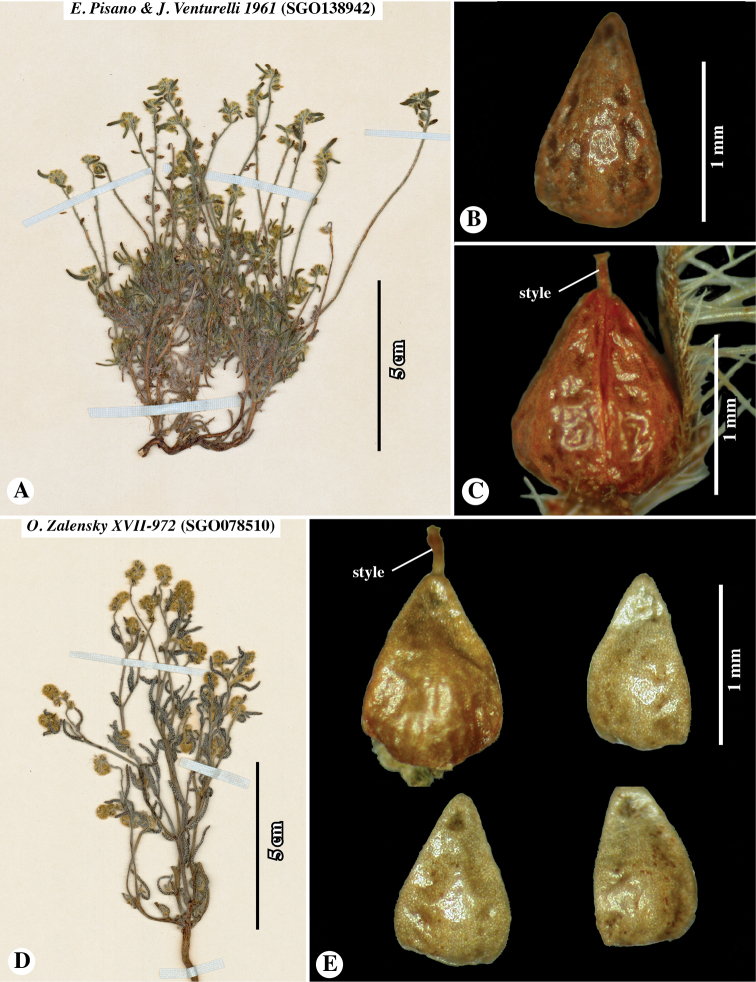
Paratypes of *Johnstonellapunensis*, sp. nov. **A–C***Pisano & Venturelli 1961* (**SGO**138942) **A** plant from herbarium sheet **B** nutlet in dorsal view, showing strongly dimpled surface **C** fruit showing four homomorphic nutlets and elongate style **D–E***Zalensky XVII-972* (**SGO**078510) **D** plant from herbarium sheet **E** four nutlets of single fruit, dorsal view; note dimpled surface and slight heteromorphism, larger, more adherent nutlet at upper left.

**Figure 6.**
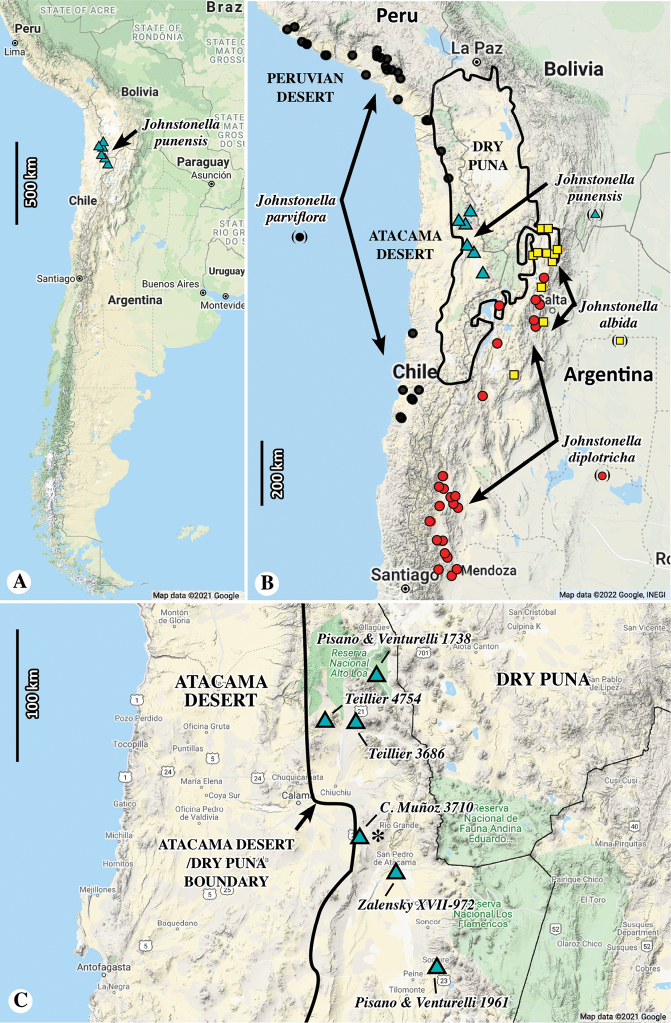
**A** distribution map of six known type collections of *Johnstonellapunensis* in South America, endemic to north-eastern Chile **B** distributions of *J.punensis* and of the other three species of South American *Johnstonella*, the latter derived from GBIF.org (2022a, b, c) data; pertinent biogeographic regions of Luebert (2021) are overlain; note restriction of *J.punensis* to the western dry Puna Region **C** close-up of distribution of *J.punensis*, showing localities of six known collections (* = holotype locality); note boundary between Atacama Desert and dry Puna; all maps from Google 2021, INEGI Data.

**Figure 7.**
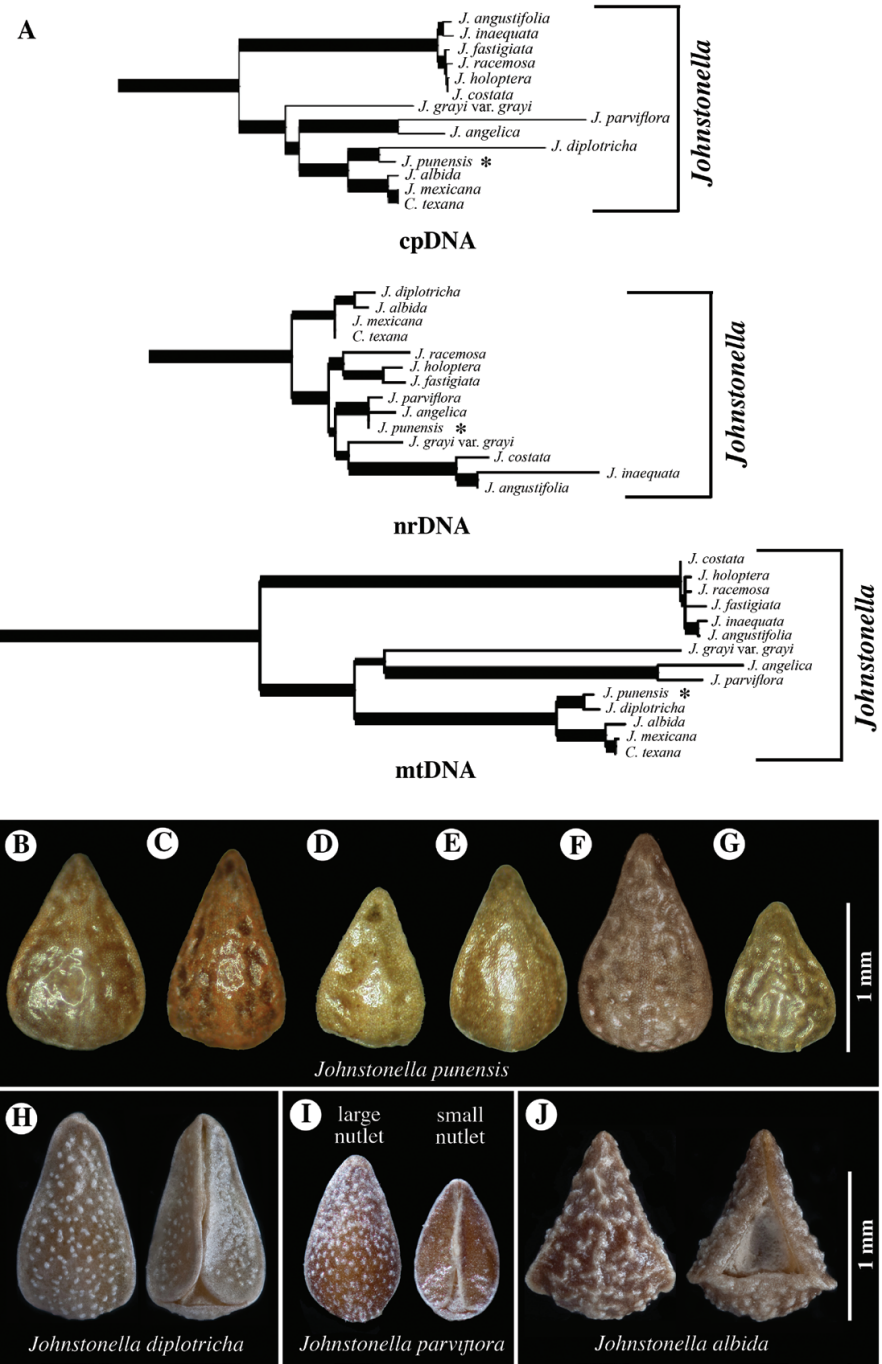
**A** Cladograms of *Johnstonella* clade from Simpson et al. 2017a, showing relationships of *Teillier 4754* (**CONC**150914) specimen of *J.punensis* (indicated with *) from analyses using sequence data of chloroplast DNA (cpDNA), nuclear ribosomal DNA (nr DNA) and mitochondrial DNA (mtDNA); note that *J.punensis* and *J.diplotricha* are sister taxa in the cpDNA and mtDNA analyses, but that *J.punensis* forms a polytomy with *J.parviflora* and *J.angelica* in the nrDNA analysis **B–J** comparison of nutlet morphology of *Johnstonella* species occurring in South America, all shown at the same scale of magnification **B–G***Johnstonellapunensis*, nutlets dimpled to rugulose, specimen source cited **B***C. Muñoz 3710* (**SGO**119165) **C***Pisano & Venturelli 1961* (**SGO**138942) **D***Zalensky XVII-972* (**SGO**078510) **E***Teillier 4754* (**CONC**150914) **F***Teillier 3686* (**SGO**139488) **G***Pisano & Venturelli 1738* (**SGO**139111); **H***Johnstonelladiplotricha*, nutlets white-tuberculate, generally homomorphic, *Haene 1779* (**SI**47823) **I***Johnstonellaparviflora*, nutlets white-tuberculate, heteromorphic with one larger (left in dorsal view) and three smaller (one of three at right, ventral view), *M. Muñoz 2715* (MO4317600) **J***Johnstonellaalbida*, nutlets coarsely tuberculate, attachment scar deep, appearing excavated, *Kiesling 3589* (**SI**87780).

Figs 1 , 2 , 3 , 4 , 5 , 6 , 7 (Note: all cited herbarium specimens indicate herbarium accession numbers)

##### Type.

Chile. Prov. de Antofagasta, Entre Calama y San Pedro de Atacama, Anual, abundante, Expedición al Desierto. 22°41.27'S, 68°28.46'W [estimated from label locality data], 2800 m elevation. 3 January 1944, *C. Muñoz 3710* (***holotype*: SGO**119165!)

##### Diagnosis.

*Johnstonellapunensis* resembles *J.diplotricha* and *J.parviflora* in having nutlets that are marginally sharp-angled, but differs in having nutlets lacking a lineate-rimmed margin and in having a surface that is dimpled to rugulose, lacking tubercles.

##### Description.

Plants annual herbs, base of plant sometimes woody at maturity, 10–15 cm tall. Root a taproot, not reddish. Stems with primary axis giving rise to secondary branches from base and mid-region, densely appressed-strigose only or appressed-strigulose and spreading to inclined-hispidulous, the trichomes whitish to greyish, 0.5–1.1 mm long. Leaves alternate, sessile, conduplicate, often recurved, grey-green, 8–13 × 1–2 mm, smaller above and at extreme base, linear to narrowly oblanceolate, entire, apex obtuse to rounded, both surfaces short-hirsute, trichomes ascending, basally white-pustulate on adaxial surface. Inflorescence of ca. 10–20 cymules, terminating upper lateral branches, straight at maturity, cymules 3–7 cm long in fruit, with ca. 10–20 flowers, peduncles 1–2 cm long, fruits erect to ascending, lowest fruits not touching, inflorescence bracts at cymule base and along peduncles, bracts similar to, slightly smaller than vegetative leaves. Flowers mostly, but not all, bracteate, bracts linear to narrowly elliptic, ascending, slightly conduplicate, straight to incurved, 3–10 mm long, decreasing in size towards apex. Pedicels ca. 0.5 mm long. Calyx ovoid, symmetric, ca. 2 mm long in flower, 2.5–3 mm long in fruit, deciduous at maturity, aposepalous, sepals lanceolate, apically narrowly acute, ascending to erect, straight to slightly recurved apically, mid-rib abaxially slightly thickened, margins appressed-hirsute, mid-rib inclined to spreading-hispid. Corolla white, rotate to funnelform, tube as long as calyx, limb ca. 1 mm broad. Gynobase 1.1–1.4 mm long, ca. as long as nutlet. Style ca. 0.5 mm long, extending 0.3–0.5 mm beyond nutlet apices. Nutlets 4, brown, erect, 1.1–1.5 × 0.7–0.9 mm, homomorphic or slightly heteromorphic in size only with the abaxial nutlet slightly larger and more adherent to the gynobase, all nutlets brown, generally ovate (length:width ratio ca. 1.3–1.8), very rarely lance-ovate, base rounded, margins sharp-angled but lacking a prominent lineate rim, apex acute, rounded at extreme tip, abaxially convex, adaxially concave-incurved, lacking papillae or tubercles, surface nearly smooth to dimpled or rugulose, spinal ridge absent, attachment scar ventral groove margins abutted or with one side slightly overlapping in upper two-thirds, with an open triangular areole in the lower third, margins laterally bifid at base.

##### Distribution and habitat.

*Johnstonellapunensis* is endemic to Chile, ranging in elevation from ca. 2800 to 3420 m. It occurs in the south-western dry Puna region near the eastern margin of the Atacama Desert (biogeographic region after Luebert 2021; see Fig. 6).

##### Phenology.

Based on data from available specimens, *Johnstonellapunensis* is reported to flower in January or June, the flowering time presumed to be dependent on precipitation.

##### Rarity and conservation status.

*Johnstonellapunensis* is known from only six collections to date. Known populations range from near La Taira (just south of Reserva Nacional Alto Loa) south to near Cuadrilla Díaz (just southwest of Parque Nacional Llullaillaco). Based on the paucity of specimens currently known, the species is likely to be deemed Data Deficient, according to guidelines of the IUCN (2022). However, we suspect that this species may qualify as a species of elevated conservation concern (IUCN 2022), because of its relatively narrow geographic range and limited known population sizes.

##### Etymology.

The specific epithet *punensis* means “of the *Puna*” (the word *puna* derived from Spanish via Quechua, the language of the aboriginal people of that region; Merriam-Webster.com 2022). The epithet highlights the restriction of this new species to the dry Puna biogeographic region (after Luebert 2021; Fig. 6).

##### Paratypes (arranged chronologically).

Chile. Prov. de Antofagasta, Ascotán, suelos arenosos, graníticos o volcánicos, crece bajo las rocas en lugares protegidos del viento, 21°32.87'S, 68°19.61'W [estimated from label locality data], 3970–4200 m elevation, 23–24 January 1943, *E. Pisano & J. Venturelli 1738* (**SGO**139111!). Prov. de Antofagasta, Socaire, suelo arenoso, en lugares secos entre las piedras, 23°35.42'S, 67°53.49'W [estimated from label locality data], 3000 m elevation, 22 February 1943, *E. Pisano & J. Venturelli 1961* (**SGO**138942!). Prov. de Antofagasta, Depto. El Loa, Valle río Vilama en las pendientes pedregosas en el desierto, 22°55.21'S, 68°11.43'W [estimated from label locality data], 2400 m elevation [estimated from label locality data], 9 June 1968, *O. Zalensky XVII-972* (**SGO**078510!). Región de Antofagasta (II), Conchi: Planta mina El Abra, Hierba perenne, flores blancas. 21°51.82'S, 68°43.11'W [estimated from label locality data], 3100 m elevation, 8 June 1995, *S. Teillier 3686* (**SGO**139488!). Región de Antofagasta (II), Camino Calama-Ascotán Volcán Poruña, Hierba anual, flores blancas, 21°53'S, 68°30'W, 3420 m elevation, 17 June 2000, *S. Teillier 4754* (**CONC**150914!) [Note: DNA was extracted from leaf material of this specimen for the studies of Simpson et al. (2017a) and Mabry and Simpson (2018)].

### ﻿Key differentiating *Cryptantha* from *Johnstonella* and separating the species of *Johnstonella* that occur in South America. Note that *J.albida* occurs in both North and South America

**Table:** 

1	Nutlets 1–4, mostly similar in size and sculpturing, if dissimilar single different nutlet positioned towards inflorescence axis (adaxial), if similar, nutlet margins rounded to sharp-angled and winged, wings lobed; nutlet surfaces smooth or papillate and/or tubercled, if tubercled, tubercles generally not whitish	***Cryptantha***
–	Nutlets 4, mostly dissimilar in size and/or sculpturing, if dissimilar, single different nutlet positioned away from inflorescence axis (abaxial), if similar, nutlet margins rounded, sharp-angled, lineate-rimmed or narrowly winged with wings not lobed; surfaces densely or sparsely white-tubercled or glabrate, dimpled or rugulose	**2 (*Johnstonella*)**
2	Nutlet surface dimpled to rugulose, lacking tubercles	***Johnstonellapunensis***
–	Nutlet surface whitish tuberculate	**3**
3	Nutlets widely ovate to deltate, margin irregularly lineate to tuberculate, attachment scar areole extending to 2/3 nutlet length, appearing deeply excavated, Argentina, Mexico, United States	***Johnstonellaalbida***
–	Nutlets ovate, margin with a well-defined lineate rim, attachment scar areole extending to 1/2 nutlet length, moderately deep, not appearing excavated	**4**
4	Calyx in fruit ca. 1.5–2 mm long; nutlets strongly heteromorphic, tuberculate, largest ca. 1 mm long, smallest 0.6–0.8 mm long, Chile, Peru	***Johnstonellaparviflora***
–	Calyx in fruit 2–4 mm long; nutlets homomorphic or slightly heteromorphic, 1.5–2 mm long; Argentina, possibly Chile	***Johnstonelladiplotricha***

## ﻿Discussion

Based on the phylogenetic study of Simpson et al. (2017a), the relationships of *Johnstonellapunensis* are equivocal (Fig. 7A). In the analysis of both chloroplast and mitochondrial DNA sequence data, *J.punensis* is sister to the South American *J.diplotricha* (Phil.) Hasenstab & M.G.Simpson with strong support. In these two analyses, *J.diplotricha* and *J.punensis* are together sister to a clade including *J.albida*, which occurs in both North and South America. However, analysis of nuclear ribosomal DNA sequence data places *J.punensis* in a polytomy with the South American *J.parviflora* (Phil.) Hasenstab & M.G.Simpson and the North American *J.angelica* (I.M.Johnst.) Hasenstab & M.G.Simpson; these last two are sister taxa in both the chloroplast and mitochondrial analyses (Fig. 7A).

*Johnstonellapunensis* and *J.diplotricha* are similar in having homomorphic or slightly heteromorphic nutlets. The two differ in that nutlets of *J.punensis* lack tubercles, having a dimpled to rugulose surface and lack a marginal lineate rim (Fig. 7B–G). Nutlets of *J.diplotricha* are whitish tuberculate and possess a prominent marginal lineate rim (Fig. 7H). In contrast, *J.parviflora* (Fig. 7I) and the North American *J.angelica* (not illustrated) are both generally strongly heteromorphic and are, in fact, quite similar to one another in numerous features (Simpson et al. 2020); both of these species have nutlets that are whitish tuberculate and marginally sharp-angled with a lineate rim. *Johnstonellaalbida* (Fig. 7J) is rather distinctive morphologically from all other South American species of the genus in having nutlets that are coarsely tuberculate, the tubercles much larger, with a deep attachment scar areole appearing strongly excavated, the scar typically extending to ca. 2/3 to the nutlet apex.

There is some variation in nutlet size and surface sculpturing observed in *J.punensis*, with nutlet length ranging from 1.1–1.5 mm long and surface sculpturing varying from nearly smooth, being only slightly and irregularly dimpled (e.g. Fig. 7E) to prominently dimpled, with irregularly circular, recessed areas (e.g. Fig. 7B–D), to what could be described as rugulose by expansion of the recessed areas (e.g. Fig. 7F–G). We currently believe this is likely natural variation within or between populations.

All known populations of *Johnstonellapunensis* occur within what is termed the dry Puna, a biogeographic region of north-eastern Chile, south-western Bolivia and limited areas of extreme north-western Argentina (a component of the western South American Dry Diagonal; see Luebert et al. 2021). By this classification, the dry Puna is just east and northeast of the Atacama Desert of Chile. However, both the Atacama Desert and the region of the dry Puna where *J.punensis* occurs are “hyperarid” with an Aridity Index (AI) of < 0.2 (after Kimura and Moriyama 2019). Germination and growth of *Johnstonellapunensis* is dependent on sporadic precipitation associated with a relative increase of summer rainfall in the dry Puna (Luebert 2021). New botanical encounters are not rare in this transition from the desert to the Altiplano highs (e.g. Calvo et al. 2018; Moreira-Muñoz and Muñoz-Schick 2020) and we encourage botanists to continue floristic studies in this harsh but marvellous zone of Chile.

Interestingly, the other South American species of *Johnstonella* occur in biogeographic regions different from that of *J.punensis* (Fig. 6B). *Johnstonellaparviflora* is restricted to Chile and Peru in the Atacama and Peruvian desert regions. *Johnstonelladiplotricha* is largely restricted to Argentina in the Prepuna Region. Finally, populations of the more distantly related *Johnstonellaalbida*, which has an American amphitropic disjunction (Guilliams et al. 2017; Simpson et al. 2017b) are restricted to Argentina, largely between the dry Puna, Monte and possibly Prepuna Regions (Luebert et al. 2021).

## ﻿Conclusions

This new species was originally detected from the results of molecular phylogenetic analyses, using leaf material removed from an herbarium specimen. Its placement was recognised to be unusual, based on previously published taxonomic concepts (Johnston 1927) and comparisons with herbarium specimens. Luckily, we were able to study fruiting material removed from the sequenced specimen, which allowed us to verify that it was, indeed, misidentified and appeared morphologically unique in its group. Study of additional herbarium collections led to the discovery of another five specimens of this taxon that fit the circumscription here proposed.

The naming of this new species points out that taxa new to science may be “sitting” in herbarium cabinets, waiting to be described (Bowdler 2010). Its discovery highlights the synergistic relationship of molecular phylogenetic analyses and of careful study of morphology from herbarium collections, the latter especially important for plants that are difficult to observe, or even locate, in the field.

## Supplementary Material

XML Treatment for Johnstonella punensis

## ﻿Appendix 1

**Specimens examined for comparative morphological studies of *Johnstonella* . Listed are collector and collection number, date of collection, and herbarium acronym and accession number.**

***J.albida***: *Cabezas 39*, February 1975 (**SI**87779); *Cabrera 21483*, 21 February 1971 (**LP**21483); *Harbison 1252*, 5 June 1903 (**SDSU**5489); *Kelley 1426*, 24 June 2007 (**SDSU**20612); *Kiesling 3589*, 15 March 1982 (**SI**87780); *Ventura 206*, 2 November 1975 (**SD**99139);

***J.diplotricha***: *Haene 1779*, 20 December 1997 (**SI**47823); *Guaglianone 2356*, 5 April 1989 (**SI**87772); *Cabrera 31803*, 17 February 1980 (**SI**87773); *Mabry 89*, 22 December 2014 (**SDSU**21232).

***J.parviflora***: *Arroyo 84-971*, 23 April 1984 (**CONC**146368); *Teillier 4816*, 5 July 2000 (**CONC**150821); *M. Muñoz 2715*, 19 October 1991 (MO4317600); *van der Wurff 20532*, 13 Apr 2006 (**MO**6128118); *Moreira 2483*, 17 June 2015 (**SDSU**21343); *Teillier 7968*, 14 September 2015 (**SDSU**21478); *Simpson 3908*, 20 October 2015 (**SDSU**21627); *Simpson 3914*, 21 October 2015 (**SDSU**22982); *Böhnert 447*, 11 March 2017 (**SDSU**23445).
